# DFT Study of Methylene Blue Adsorption on ZnTiO_3_ and TiO_2_ Surfaces (101)

**DOI:** 10.3390/molecules26133780

**Published:** 2021-06-22

**Authors:** Ximena Jaramillo-Fierro, Luis Fernando Capa, Francesc Medina, Silvia González

**Affiliations:** 1 Departamento d’Enginyería Química, Universitat Rovira i Virgili, Av Països Catalans, 2643007 Tarragona, Spain; francesc.medina@urv.cat; 2 Departamento de Química y Ciencias Exactas, Universidad Técnica Particular de Loja, San Cayetano Alto, 1101608 Loja, Ecuador; sgonzalez@utpl.edu.ec; 3 Maestría en Química Aplicada, Universidad Técnica Particular de Loja, San Cayetano Alto, 1101608 Loja, Ecuador; lfcapa@utpl.edu.ec

**Keywords:** DFT, ZnTiO_3_, TiO_2_, methylene blue, adsorption

## Abstract

The search for alternative materials with high dye adsorption capacity, such as methylene blue (MB), remains the focus of current studies. This computational study focuses on oxides ZnTiO_3_ and TiO_2_ (anatase phase) and on their adsorptive properties. Computational calculations based on DFT methods were performed using the Viena Ab initio Simulation Package (VASP) code to study the electronic properties of these oxides. The bandgap energy values calculated by the Hubbard *U* (GGA + U) method for ZnTiO_3_ and TiO_2_ were 3.17 and 3.21 eV, respectively, which are consistent with the experimental data. The most favorable orientation of the MB adsorbed on the surface (101) of both oxides is semi-perpendicular. Stronger adsorption was observed on the ZnTiO_3_ surface (−282.05 kJ/mol) than on TiO_2_ (–10.95 kJ/mol). Anchoring of the MB molecule on both surfaces was carried out by means of two protons in a bidentate chelating (BC) adsorption model. The high adsorption energy of the MB dye on the ZnTiO_3_ surface shows the potential value of using this mixed oxide as a dye adsorbent for several technological and environmental applications.

## 1. Introduction

Over the past decade, Ti- and Zn-based oxides have received much attention due to their competitive cost, non-toxicity, excellent stability, availability, and ability to produce highly oxidizing radicals [1,2,3,4]. Titanium oxide (TiO_2_) and zinc oxide (ZnO) are two well-known semiconductors that have been widely used to construct electron transport channels due to their appropriate bandgaps, efficient electron mobilities, and simple synthesis methods [5,6,7,8]. In addition, these oxides are promising semiconductors to eliminate organic pollutants with incomparable efficiency due to their tunable surface and structural functionality [9]. The ZnO–TiO_2_ composite system has even more superior properties than the individual oxides due to the high separation rate of photogenerated carriers and the wide optical response range [10]. Several syntheses and characterization studies of the ZnO–TiO_2_ system have shown that there are three compounds in this binary system, including ZnTiO_3_ (cubic, hexagonal), Zn_2_TiO_4_ (cubic, tetragonal), and Zn_3_Ti_3_O_8_ (cubic) [11,12,13].

ZnTiO_3_ has many similar physical properties to TiO_2_ and ZnO, including high electron mobility [14,15,16]. This ternary oxide has been widely used because of its outstanding properties and potential scientific and technical applications [17]. ZnTiO_3_ has been investigated in a variety of applications as an antibacterial, catalyst, nanofiber, white pigment, microwave dielectric, gas sensor, nonlinear optical, corrosion inhibitor, and luminescent material [18,19,20,21,22,23], but its application in adsorption has not been sufficiently studied, despite the fact that the literature indicates that due to its great specific area, it could have an important potential as an adsorbent [24,25].

ZnTiO_3_ is a polar oxide of the LiNbO_3_-type (LN-type) with both cations coordinated octahedrally in a three-dimensional framework of the octahedron perovskite (Pv) that shares corners [26]. In this structure, both cations move along the trigonal axis c, thus producing a spontaneous polarization reinforced by a second-order Jahn–Teller (SOJT) distortion due to Ti^4+^ (d^0^) [27]. The paraelectric parent structure of ZnTiO_3_ is the ilmenite (Il)-type phase (hexagonal space group *R-3*), which is the stable phase under ambient conditions [28]. The crystalline and phase transformation behaviors of ZnTiO_3_ have systematically been investigated by various authors regarding several synthesis methods, Ti:Zn precursor molar ratios, and calcination temperatures [29,30,31,32,33,34,35]. Furthermore, the literature agrees that obtaining ZnTiO_3_ as a pure phase at a low processing temperature is a challenge in materials chemistry [36,37,38].

In a previous experimental paper, we reported the synthesis and characterization of the ZnTiO_3_/TiO_2_ nanocomposite. This heterostructure was indexed to a hexagonal phase with space group R-3(148) for ZnTiO_3_ and a tetragonal phase with space group I4_1_/amd(141) for TiO_2_ (anatase) [39,40]. In these studies, we also reported the ability of ZnTiO_3/_TiO_2_ to remove the methylene blue (MB) dye in aqueous systems, and it was contrasted with the results obtained for pure TiO_2_ (anatase). The results showed that the heterostructure has better photocatalytic adsorption and degradation capacity than anatase alone, probably due to a synergistic effect. This synergistic effect between semiconductors has been extensively studied, showing that the presence of a second semiconductor can provide special active sites to enhance the adsorption and photocatalysis of various compounds [41].

Although several properties of ZnTiO_3_ have been extensively studied experimentally, a proper description of its electronic, optical, and adsorptive properties remains an active research area from a theoretical point of view [42] since, up to date, this ternary oxide has scarcely been studied with quantum methods [43]. Therefore, the computational study of the molecular interaction between ZnTiO_3_ and methylene blue could contribute to clarifying the adsorption and degradation mechanism of this dye, favoring the development of materials for the treatment of waters contaminated with MB.

The elimination of MB in wastewater is an extremely important task in environmental protection because it has caused serious contamination in many countries of the world [44]. Methylene blue, known as methylthioninium chloride is a basic cationic dye widely used in the printing, plastics, paper, leather, food, pharmaceutical, and textile industries [45,46,47]. The discharge of wastewater effluents from these industries, with a high content of MB without efficient degradation, results in harmful effects for humans and animals [48].

Various technologies have been used to treat wastewater contaminated with dyes [49]. Among these techniques, adsorption is easy to perform without pretreatment and is highly selective for removing dyes [50]. In addition, adsorption has been found to be superior to other techniques for wastewater treatment in terms of initial cost, simplicity of design, ease of operation, and insensitivity to toxic substances [24]. Although there are several experimental studies of MB adsorption on different surfaces, some uncertainties remain due to lack of understanding at the molecular level of the MB adsorption mechanism on the ZnTiO_3_ surface.

As is well known, computational calculations of the electronic structure in an isolated molecule can achieve the desired chemical precision as long as a sufficiently large basis set is used, the electronic correlation is sufficiently described, and the relativistic effects in the calculation are adequately included [51]. Therefore, in this study, Density Functional Theory (DFT) computational calculations were used to characterize the electronic structure of ZnTiO_3_ and TiO_2_ (anatase) and also to investigate the feasibility of using both oxides as MB adsorbents. The results presented in this paper clarify the previously obtained experimental results and confirm that ZnTiO_3_ is an excellent adsorbent and that it has high potential for future technological and environmental applications.

## 2. Results

### 2.1. Optimization and Electronic Structure of ZnTiO_3_ and TiO_2_

**Figure 1:**
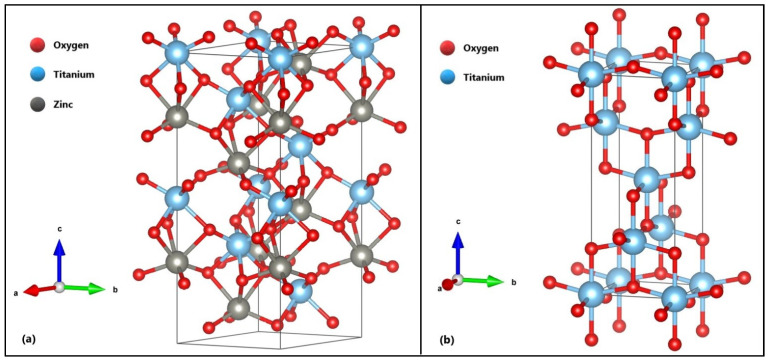
Optimized structures of (**a**) ZnTiO_3_ and (**b**) TiO_2_.

The adsorption of the methylene blue molecule on the surface of both ZnTiO_3_ and TiO_2_ was modeled using the following parameters: hexagonal ZnTiO_3_ with a cell = 5.148 Å × 5.148 Å × 13.937 Å <90° × 90° × 120°> and tetragonal TiO_2_ with a cell = 3.821 Å × 3.821 Å × 9.697 Å <90° × 90° × 90°>, as shown in Figure 1. The coordinates of the optimized ZnTiO_3_ and TiO_2_ structures are detailed in Table S1 and the corresponding optimization energy values are included in Figure S2.

**Figure 2:**
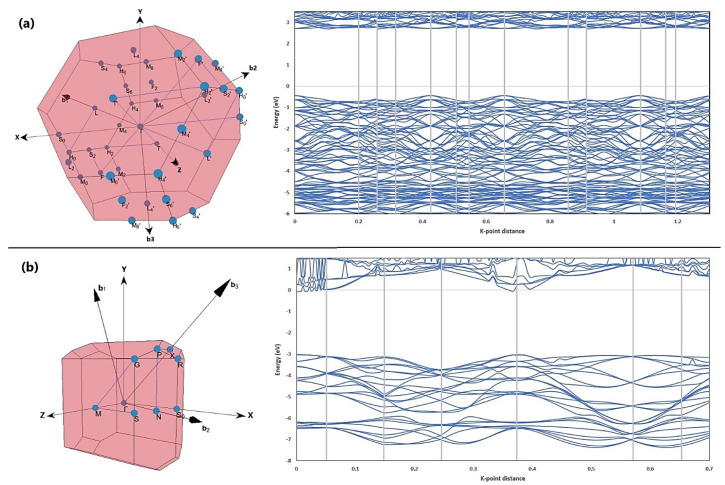
Band structures of (**a**) ZnTiO_3_ and (**b**) TiO_2_ along the high symmetry directions in the Brillouin zone.

The selection of the high symmetry points and lines in the first Brillouin zone [52] and the results of the calculation of the electronic band structure of ZnTiO_3_ and TiO_2_ are shown in Figure 2a,b, respectively.

Figure 2a,b show that the indirect bandgap energy values of the ZnTiO_3_ and TiO_2_ structures calculated by the exchange–correlation functional in the generalized gradient approximation (GGA-PBE) method were 2.20 and 2.31 eV, respectively. However, the indirect bandgap values were also calculated by the GGA + U method, that is, incorporating the Hubbard *U* approximation term. Indirect bandgap calculations using GGA + U resulted in 3.16 eV (U = 2.5) and 3.21 eV (U = 4.0) for ZnTO_3_ and TiO_2_, respectively. These results are in good agreement with the experimental results reported in the literature: 3.18 eV for ZnTiO_3_ [15] and 3.20 eV for TiO_2_ [53].

**Figure 3:**
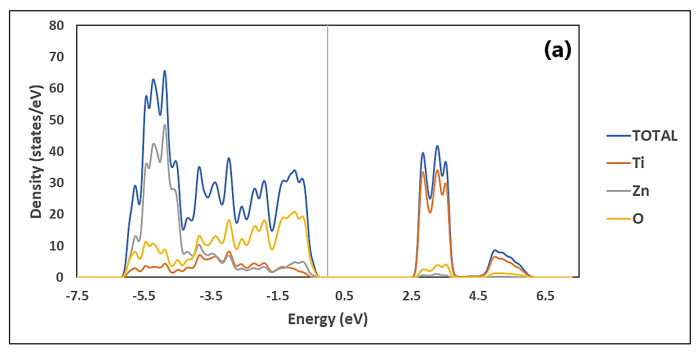

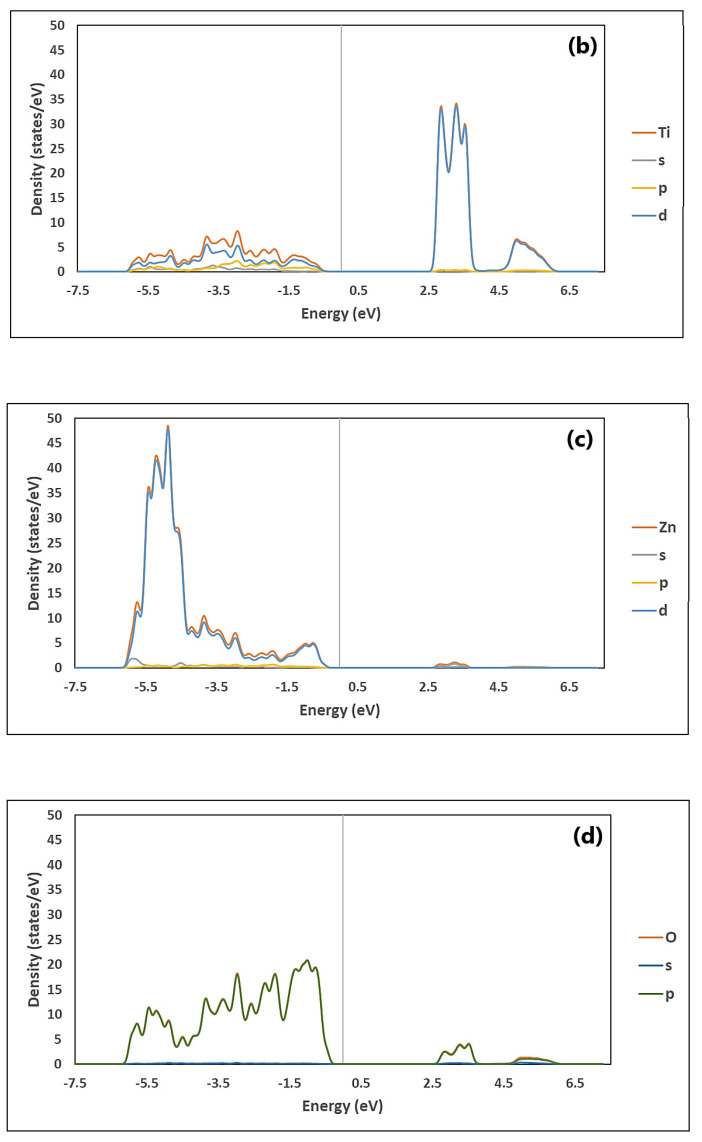
Density of states (DOSs) of ZnTiO_3:_ (**a**) total, and partial: (**b**) Ti, (**c**) Zn and (**d**) O.

**Figure 4:**
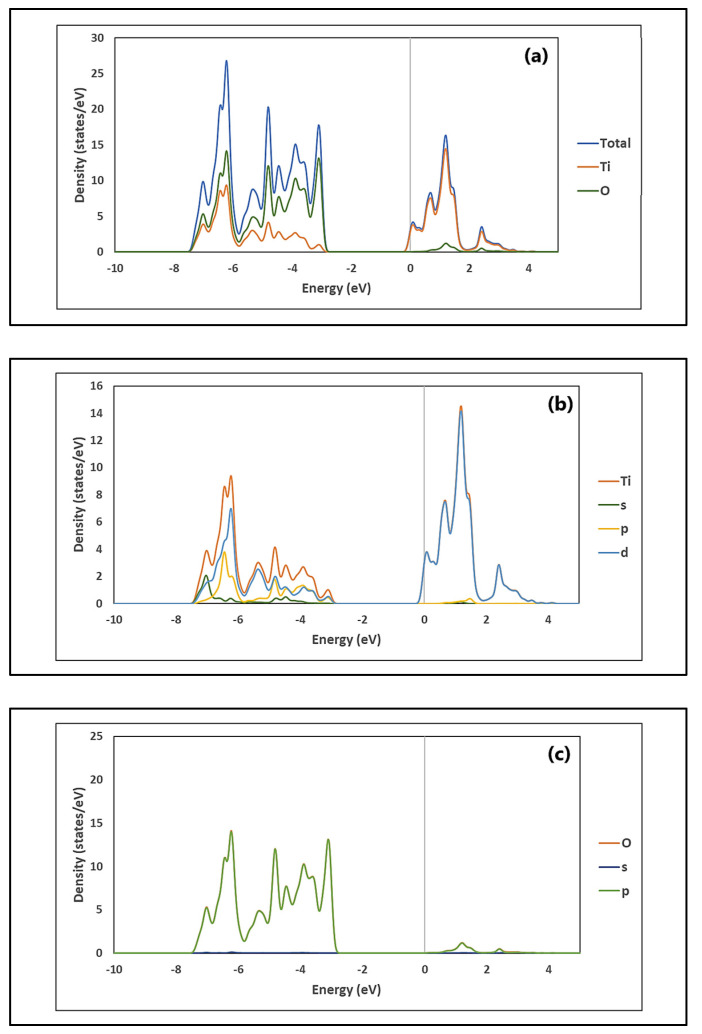
Density of states (DOSs) of TiO_2_: (**a**) total, and partial: (**b**) Ti and (**c**) O.

The total and partial density of states (DOS) of ZnTiO_3_ and TiO_2_ are illustrated in Figure 3 and Figure 4, respectively. Figure 3a shows that the total density of state (TDOS) of ZnTiO_3_ has two main zones: an upper conduction band (CB) zone from 2.5 to 6.2 eV, and a lower valence band (VB) zone, from −6.0 to −0.2 eV. The CB is dominated by the contribution of Ti, while the VB is dominated by the contribution of Zn and O. Figure 3b–d show the partial density of state (PDOS) of ZnTiO_3_. As can be seen in Figure 3b, the main contribution of Ti in the CB is through the 3*d* orbital. On the other hand, Figure 3c shows that Zn contributes mainly to the VB through the 3*d* orbital while O interacts with Zn in this band through the 2*p* orbital shown in Figure 3d.

Likewise, Figure 4a shows that the total density of state (TDOS) of TiO_2_ has two main zones: an upper conduction band (CB) zone from −0.1 to 3.8 eV, and a lower valence band (VB) zone, from −7.6 to −2.8 eV. The CB is dominated by the contribution of Ti, while the VB is dominated by the contribution of O. Figure 4b,c show the partial density of state (PDOS) of TiO_2_. As can be seen in Figure 4b, the main contribution of Ti in the CB is through the 3*d* orbital. On the other hand, Figure 4c shows that O contributes mainly to the VB through the 2*p* orbital.

In both structures, ZnTiO_3_ and TiO_2_, the valence band maximum (VBM) is bordered by the oxygen atom, while the Ti atom determines the conduction band maximum (CBM). Consequently, ZnTiO_3_ has an energy bandgap quite similar to that of TiO_2_, due to the fact that ZnTiO_3_ involves both ZnO and TiO_2_. Our calculated results agree with the literature [5,18,54].

In order to further understand the chemical bonding of hexagonal ZnTiO_3_ and tetragonal TiO_2_, the population analyses were estimated by the Bader method. For ZnTiO_3_, the net charge of Ti (+2.6*e*) was 1.4*e*, much smaller than its +4*e* formal charge, whereas the Zn atom had a positive charge of +1.4*e* and the O atom had a negative charge of −1.3*e*, which are less than their +2*e* and −2*e* formal charges by 0.6*e* and 0.7*e*, respectively. For TiO_2_, the net charges of the Ti and O atoms were similar to those calculated for the Ti and O atoms of ZnTiO_3_. These results agree with those reported by other authors [55]. Since the charges on the different bonds can reflect the covalent and ionic properties of the molecule, we concluded that the Ti–O bond is typically covalent for both ZnTiO_3_ and TiO_2_ and that the Zn–O bond for ZnTiO_3_ is typically ionic; these results coincide with those reported in the literature [42,56].

### 2.2. Adsorption of the MB Dye on the Structures

**Figure 5:**
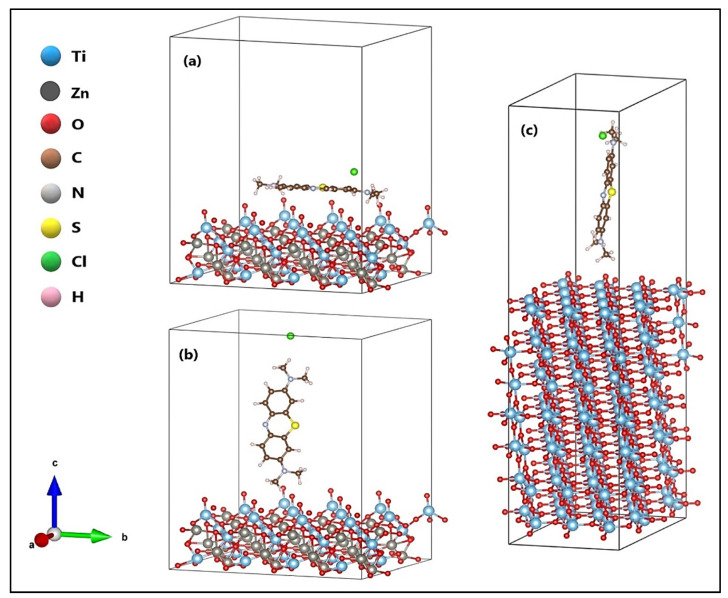
Methylene blue (MB) molecule in (**a**) horizontal and (**b**) semi-perpendicular orientation on the ZnTiO_3_ surface, and (**c**) semi-perpendicular orientation on the TiO_2_ surfaces.

The orientations of the MB molecule on the ZnTiO_3_ and TiO_2_ surfaces are shown in Figure 5. Figure 5a shows the horizontal orientation of the MB molecule on the ZnTiO_3_ surface, while Figure 5b,c show the semi-perpendicular orientation of the MB molecule on the ZnTiO_3_ and TiO_2_ surfaces, respectively. The adsorption of the MB molecule on the ZnTiO_3_ surface with the molecule placed in semi-perpendicular orientation (E_ads_ = −2.916 eV) was more energetically favored than in the horizontal orientation (E_ads_ = −1.310 eV). Therefore, we studied the adsorption of MB on the TiO_2_ surface only with the semi-perpendicular orientation. The calculated adsorption energy for the TiO_2_ surface (E_ads_ = −0.113 eV) was less favorable than the calculated adsorption energy for the ZnTiO_3_ surface.

**Figure 6:**
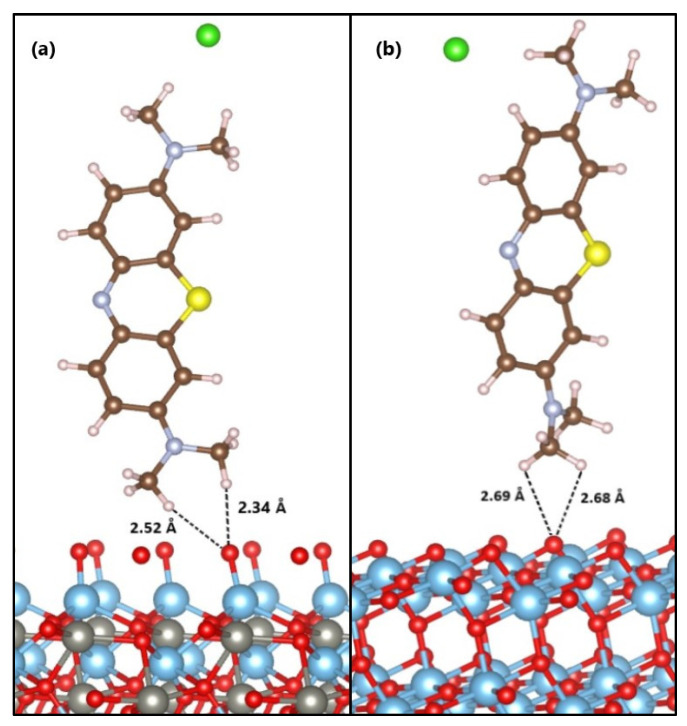
Anchoring modes of the MB molecule on the surface of (**a**) ZnTiO_3_ and (**b**) TiO_2_.

The anchoring modes of the MB molecule on the ZnTiO_3_ and TiO_2_ surfaces are shown in Figure 6. Adsorption of the dye on the ZnTiO_3_ and TiO_2_ surfaces occurs in a bidentate chelating (BC) adsorption model [57] with two protons oriented toward the nearest surface oxygen [58].

The calculated adsorption energy value indicates that the MB molecule is strongly adsorbed on the ZnTiO_3_ surface. The average distances from the hydrogen atoms of the MB molecule (H_MB_) to the surface plane of ZnTiO_3_ are O_(oxide)_-H_MB_ = 2.34 Å and O_(oxide)_-H_MB_ = 2.52 Å. Moreover, the adsorption energy value of the MB molecule on the TiO_2_ surface indicates a weaker interaction than on ZnTiO_3_ (E_ads_ = −0.113 eV). The average distances from the hydrogen atoms of the molecule to the plane of the TiO_2_ surface are O_(oxide)_-H_MB_ = 2.68 Å and O_(oxide)_-H_MB_ = 2.69 Å.

## 3. Discussion

### 3.1. Optimization and Electronic Structure of ZnTiO_3_ and TiO_2_

The description of the electronic structure of materials involving transition metals with DFT is often complicated due to correlation effects involving 3*d* electrons [59]. However, since the transition metals of the oxides studied in this paper are formally in the 3*d*^0^ or 3*d*^10^, neither TiO_2_ nor ZnTiO_3_ are strongly correlated materials; consequently, a simple DFT approach allowed us to accurately calculate the conduction and valence bands of ZnTiO_3_ and TiO_2_.

The literature shows that ZnTiO_3_ has a relatively wide bandgap (E_g_ = 2.73–3.70 eV), the value of which depends on the synthesis conditions [60,61]. In our study, the ZnTiO_3_ and TiO_2_ structures presented indirect bandgap values of 2.20 and 2.31 eV, respectively, which were calculated by the GGA-PBE method. In contrast with the experimental data, 3.18 eV for ZnTiO_3_ [15] and 3.20 eV for TiO_2_ [53], the theoretical values are lower, and this may be due to the widely known DFT-underestimation of the bandgap in most materials [1,15]. Therefore, a Hubbard *U* approximation term was adopted to accurately describe the electronic structures [12,62]. The new indirect bandgap values calculated by GGA + U were 3.16 and 3.21 eV for ZnTiO_3_ and TiO_2_, respectively, which are consistent with the aforementioned experimental data. As can be seen, mixed oxide ZnTiO_3_ has lower bandgap energy than TiO_2_. According to the literature, this occurs due to the replacement of Ti (3*d*^0^) atoms with Zn (3*d*^10^) that induce O 2*p*-Zn 3*d*^10^ repulsion [21,63]. Table 1 shows the comparison of the bandgap energy values of the ZnTiO_3_ and TiO_2_ calculated in this study with other energy values reported in the literature.

The main character of the electronic structure of ZnTiO_3_ originates mainly from the hybridization between the Ti-3*d* and O-2*p* states. The Zn-3*d* and O-2*p* hybridization and the Ti-3*d* and O-2*p* hybridization as well as nonbonding O-2*p* states are observed at the upper valence bands (VBs). The localized Zn-3*d* states indicate weak Zn-3*d* and O-2*p* hybridization. The states at the lower conduction bands (CBs) are attributed to antibonding states from Ti-3*d* and O-2*p*. These results agree with those reported by other authors [42]. Similarly, the main character of the electronic structure of TiO_2_ originates from the hybridization between the Ti-3*d* and O-2*p* states. This hybridization, as well as nonbonding O-2*p* states, is observed at the upper valence bands (VBs), while the states at the lower conduction bands (CBs) are attributed to antibonding states from Ti-3*d* and O-2*p*. In both structures, the lowest-energy states are due to the isolated Ti-3*s*, Ti-3*p*, and O-2*s* states. These results also agree with those reported by other authors [5,54,65].

### 3.2. Adsorption of the MB Dye on the Oxide Models

Several experimental studies of MB removal in aqueous systems have shown that this cationic dye can be easily adsorbed on the ZnTiO_3_ and TiO_2_ surfaces, due to electrostatic attraction [49]. Therefore, the surface oxygen atoms of both oxides probably generate negatively charged sites that easily attract positive regions of the MB molecule, favoring molecular adsorption.

Our results indicated that the semi-perpendicular orientation of the MB molecule with respect to both oxide surfaces is more favored. In fact, the MB molecule oriented parallel to the surface shows a strong preference for the methyl group of the molecule, while the aromatic ring bends slightly away from the surface due to electrostatic repulsion between the N and S atoms from the aromatic ring and surface oxygen. This is consistent with several studies reporting good adsorption results for dye molecules oriented perpendicular to the adsorbent surface [57,66,67]. Greathouse et al. mentioned that at very high concentrations, this dye forms aggregates that are adsorbed vertically to the surface [68]. Our results suggested that this orientation of the MB molecule on the surface is caused by the balance between electrostatic repulsion between adjacent ions and the strong hydrophobic MB–MB and MB–surface interactions [69].

The MB molecule was adsorbed on the ZnTiO_3_ surface (101) with higher negative energy (E_ads_ = −2.92 eV) than on the TiO_2_ surface (101) (E_ads_ = −0.12 eV), and the average adsorption distances (O–H) were 2.43 and 2.68 Å for ZnTiO_3_ and TiO_2_, respectively. According to the optimized configurations, in both cases, the approach is from the two H atoms of the methyl group of the MB molecule to an O atom of each surface. In agreement with the literature, hydrogen bonding can increase the stability of the interaction of a dye with the ZnTiO_3_ and TiO_2_ surfaces during the adsorption process [70,71]. The Bader charge analysis in Table S1 indicates no significant electronic exchange between the MB molecule and the oxides surface [72]. The results obtained in this theoretical study suggest that MB adsorption is more stable (higher negative adsorption energy) on the ZnTiO_3_ surface than on the TiO_2_ surface, which is consistent with previous experimental studies.

Pastore et al. suggested three typical coordination schemes: monodentate, bidentate chelating, and bidentate bridging [57]. In our study, we found that the MB molecule is adsorbed on the ZnTiO_3_ and TiO_2_ surfaces (101) in a bidentate chelating mode, which, according to several authors, produces more stable adsorption with more exothermic adsorption energy [70,73]. The shape of the dye molecules anchored to the oxide significantly affects the molecular adsorption energy. Therefore, calculating the adsorption energy value gives insight into the adherence strength and shape of molecule–surface bonding [66]. The higher the adsorption energy, the higher the retention of the dye on the oxide surface, this being a desirable condition to apply to subsequent photosensitive processes.

In the literature, not enough computational studies of MB adsorption on semiconductors were found; consequently, in Table 2, the results obtained in the present study are compared with those results reported in the literature for the adsorption of other dyes on ZnTiO_3_ (101) and TiO_2_ (101).

Other research studies should analyze and apply these results to implement adsorptive systems for dyes or similar molecules.

## 4. Materials and Methods

All Density Functional Theory (DFT) calculations were performed using the Viena Ab initio Simulation Package (VASP), version 5.3.3 [15,74]. The Perdew–Burke–Ernzerhof (PBE) exchange–correlation functional in the generalized gradient approximation (GGA) proposed by Perdew et al. [75] was employed. The augmented plane wave (PAW) method was used to describe the electron–ion interactions [15]. The cutoff energy to the plane waves was set to 500 eV. The Kohn–Sham equations [76,77] were solved self-consistently until the energy variation between cycles was less than 10^−5^ eV. The first Brillouin zone was sampled using Monkhorst–Pack [78] *k*-point meshes to calculate the bulk properties of ZnTiO_3_ and TiO_2_, in particular, 3 × 7 × 5 and 3 × 3 × 1, respectively. All atomic positions were fully relaxed until the forces on each atom were below 0.01 eV/Å. The computational parameters were selected seeking the best balance between computational cost and precision. The tested values were as follows: energy cutoff points = 450, 475, 500, and 515 eV; force convergence criterion for ionic relaxation = 0.08, 0.04, 0.02, 0.01, and 0.005 eV/Å and number of *k-*points corresponding to *k*-spacing in each axe = 0.35, 0.30, 0.25, 0.20, and 0.15. The parameters were optimized until the difference between the energy values of the system was lower than 10^−4^ eV.

The Gaussian smearing method with σ = 0.10 eV was applied to band occupations in order to improve total energy convergence [15]. A Hubbard *U* approximation term was adopted to describe the strong on-site Coulomb repulsion in order to accurately explain the electronic structures [62], which is not correctly described by the PBE functional [58]. Population analyses were estimated using Bader’s charge analysis code, which provided important information on bonding behaviors from the atomic charge values [79,80,81]. All calculations were non-spin polarized and all molecular models were created and visualized using BioVia Material Studio, version 5.5.

To study MB adsorption, an optimized molecular structure was used [68]. The bulk of both ZnTiO_3_ and TiO_2_ crystals was cleaved on the surface (101), since it is the most stable surface according to the literature [54,57,62,66,82]. The slab model of ZnTiO_3_ (101) was a supercell p(2 × 3) with three atomic layers, which includes 36 Zn atoms, 36 Ti atoms, and 108 O atoms. On the other hand, the TiO_2_ (101) surface model has seven atomic layers with a p(3 × 3) structure of the original unit cell, which includes 168 Ti atoms and 336 O atoms.

An appropriate vacuum thickness of each structure was chosen by calculating the surface energy. For both ZnTiO_3_ and TiO_2_ surface models, a vacuum of 20 Å was added. The surface energies (*γ_s_*) were calculated using the following equation [83]:

(1) $$\gamma_{s}=\frac{\left(E_{slab}-n\times E_{bulk}\right)}{2A}$$

where *E_slab_* is the total energy of the slab material (eV), *E_bulk_* is the total energy of the bulk material (eV), *n* is the number of atoms contained in the slab, and *A* is the surface area (Å^2^). The values for the surface energies (γ_s_) of the ZnTiO_3_ and TiO_2_ structures with a vacuum distance of 20 Å were 0.076 eV/Å^2^ (7.30 kJ/Å^2^) and 0.062 eV/Å^2^ (5.98 kJ/Å^2^), respectively.

Adsorption calculation was initiated with the MB molecule placed close to the surface of each oxide in at least one of the following orientations, horizontal (H) and semi-perpendicular (SP), with respect to the surface.

The adsorption energy (Δ*E_ads_*) of the MB molecule on the surface of both ZnTiO_3_ and TiO_2_ oxides was calculated using the following equation [84]:

(2) $$\Delta E_{ads}=E_{MB/oxide}-E_{oxide}-E_{MB}$$

where *E_MB_*_/*oxide*_ is the energy of the supersystem formed by the adsorbed molecule on the surface (eV), *E_oxide_* is the energy of the clean oxide (eV), and *E_MB_* is the energy of the isolated molecule in vacuum (eV).

## 5. Conclusions

The aim of this comparative study was to use molecular simulation to address unresolved issues related to the adsorption mechanism of methylene blue on both ZnTiO_3_ and TiO_2_. DFT calculations of MB adsorption on the surface (101) of both ZnTiO_3_ and TiO_2_ indicated that adsorption on ZnTiO_3_ was stronger than on TiO_2_. The semi-perpendicular orientation was the most probable molecular approach to the oxide surfaces. Electrostatic repulsion due to the proximity of adjacent S and N atoms when MB was in high concentration was overcome by the much stronger interactions between the methyl groups and the surface oxygen atoms of ZnTiO_3_ and TiO_2_.

Finally, we computationally corroborated the feasibility of using ZnTiO_3_ as an MB adsorbent material, as experimentally found. Theoretically, we forecast the appealing prospect for this material according to the adsorption energy and the large bandgap calculated by DFT, which is in addition to the experimental results that we reported in a previous paper. Our study verifies that ZnTiO_3_ has better MB adsorption energy than TiO_2_ in the anatase phase, which is important to enhance a subsequent degradation process. The large bandgap obtained by DFT calculations also shows that ZnTiO_3_ can potentially be used as a photocatalyst, allowing for complete degradation of the dye after being adsorbed. Therefore, considering only the band structure, ZnTiO_3_ fully meets the necessary requirements to be a photocatalyst. As already mentioned, however, in addition to the band structure, the adsorption capacity is also very important for photocatalytic materials. In this way, ZnTiO_3_ constitutes an efficient alternative material for various technological and environmental applications.

## Figures and Tables

**Table 1:** Calculated bandgap energy of ZnTiO_3_ and TiO_2_ and other values reported in the literature.

Adsorbent	Software Used	Basis Set Used/Functional Used	Bandgap (eV)	Reference
ZnTiO_3_	CASTEP	GGA/SP-PBE	3.14	[1]
ZnTiO_3_	CASTEP	GGA+U	3.28	[1]
ZnTiO_3_	MS-DMol3	GGA/PBE	3.10	[5]
ZnTiO_3_	MS-DMol3	GGA/PPE-grime	3.53	[5]
ZnTiO_3_	MS-DMol3	GGA/PPE-TS	3.12	[5]
ZnTiO_3_	Experimental		3.18	[15]
ZnTiO_3_	VASP	GGA/PBE	2.96	[15]
ZnTiO_3_	CASTEP	GGA/PW91	3.47	[18]
ZnTiO_3_	ABINIT	HSE06	4.25	[56]
ZnTiO_3_	ABINIT	GGA/NC	3.25	[56]
ZnTiO_3_	ABINIT	LDA/NC	3.05	[56]
ZnTiO_3_	ABINIT	GGA/ultrasoft	2.96	[56]
ZnTiO_3_	ABINIT	LDA/ultrasoft	2.86	[56]
ZnTiO_3_	VASP	GGA/PBE+U	3.16	This study
ZnTiO_3_	VASP	GGA/PBE	2.20	This study
TiO_2_	VASP	HSE06	3.20	[62]
TiO_2_	VASP	GGA/PBE	2.55	[58]
TiO_2_	VASP	GGA/PBE+U	3.11	[58]
TiO_2_	Experimental		3.20	[53]
TiO_2_	CASTEP	GGA/PBE	2.70	[53]
TiO_2_	CASTEP	GGA/PBE+U	3.34	[53]
TiO_2_	ABINIT	GGA/PBE	2.08	[64]
TiO_2_	ABINIT	GW	3.71	[64]
TiO_2_	VASP	GGA/PBE	2.31	This study
TiO_2_	VASP	GGA/PBE+U	3.21	This study

**Table 2:** Calculated adsorption energy of ZnTiO_3_ and TiO_2_ and other values reported in the literature.

Adsorbent	Dye	Software Used	Basis Set/Functional Used	Adsorption		References
				eV	kJ/mol	
ZnTiO_3_ (101)	TPA-1	CASTEP	GGA/PBE	−1.41	−136.39	[66]
ZnTiO_3_ (101)	TPA-2	CASTEP	GGA/PBE	−1.63	−157.47	[66]
ZnTiO_3_ (101)	TPA-3	CASTEP	GGA/PBE	−5.82	−561.33	[66]
ZnTiO_3_ (101)	TPA-4	CASTEP	GGA/PBE	−2.37	−228.19	[66]
ZnTiO_3_ (101) (H)	MB	VASP	GGA/PBE	−1.31	−126.76	This study
ZnTiO_3_ (101) (SP)	MB	VASP	GGA/PBE	−2.92	−282.05	This study
TiO_2_ (101)	R4-BT	VASP	GGA/PBE	−1.40	−135.46	[58]
TiO_2_ (101)	R4-F2BT	VASP	GGA/PBE	−1.39	−134.50	[58]
TiO_2_ (101)	R4-BO	VASP	GGA/PBE	−1.39	−134.50	[58]
TiO_2_ (101)	R6-Bz	VASP	GGA/PBE	−1.40	−135.46	[58]
TiO_2_ (101)	R6-BT	VASP	GGA/PBE	−1.38	−133.53	[58]
TiO_2_ (101)	R6-F2BT	VASP	GGA/PBE	−1.37	−132.56	[58]
TiO_2_ (101)	R6-B0	VASP	GGA/PBE	−1.37	−132.56	[58]
TiO_2_ (101)	R6-Bz	VASP	GGA/PBE	−1.38	−133.53	[58]
TiO_2_ (101) (SP)	MB	VASP	GGA/PBE	−0.12	−11.61	This study

## Data Availability

Data are contained within the article and Supplementary Materials.

## Supplementary Materials

The following are available online. Figure S1: Aromatic ring of MB bent slightly on the ZnTiO_3_ surface (101); Figure S2: Optimization energies of ZnTiO_3_, TiO_2_ and MB; Table S1: Coordinates of the optimized structures; Table S2: Bader’s charge analysis of the methylene blue molecule.

## Sample Availability

Samples of the compounds are not available from the authors.
