# Supplementary material for: DFT Study of Methylene Blue Adsorption on ZnTiO3 and TiO2 Surfaces (101)
Source: Molecules. 2021 Jun 22;26(13):3780. doi: 10.3390/molecules26133780 (PMC8270277; doi:10.3390/molecules26133780)
Supplement: Supplementary file 1 [file molecules-26-03780-s001.zip › molecules-12531640-supplementary.pdf]

molecules

Supplementary Material

DFT Study of Methylene Blue Adsorption on $ZnTiO_3$ and TiO_2 Surfaces (101)

Ximena Jaramillo-Fierro [1,2,*], Luis Fernando Capa [3], Francesc Medina [4] and Silvia González [2]

1 Departamento d'Enginyería Química, Universitat Rovira i Virgili, Av Països Catalans, 2643007 Tarragona, Spain
2 Departamento de Química y Ciencias Exactas, Universidad Técnica Particular de Loja, San Cayetano Alto, Loja 1101608, Ecuador; sgonzalez@utpl.edu.ec
3 Maestría en Química Aplicada, Universidad Técnica Particular de Loja, San Cayetano Alto, Loja 1101608, Ecuador; lfcapa@utpl.edu.ec
4 Departamento d'Enginyería Química, Universitat Rovira i Virgili, Av Països Catalans, 2643007 Tarragona, Spain; francesc.medina@urv.cat
* Correspondence: xvjaramillo@utpl.edu.ec; Tel.: +593-7-3701444

Abstract: The search for alternative materials with high dye adsorption capacity, such as methylene blue (MB), remains the focus of current studies. This computational study focuses on oxides $ZnTiO_3$ and TiO_2 (anatase phase) and on their adsorptive properties. Computational calculations based on DFT methods were performed using the Viena Ab initio Simulation Package (VASP) code to study the electronic properties of these oxides. The bandgap energy values calculated by the Hubbard *U* (GGA+U) method for $ZnTiO_3$ and TiO_2 were 3.17 and 3.21 eV, respectively, which are consistent with the experimental data. The most favorable orientation of the MB adsorbed on the surface (101) of both oxides is semi-perpendicular. Stronger adsorption was observed on the $ZnTiO_3$ surface (−282.05 kJ/mol) than on TiO_2 (−10.95 kJ/mol). Anchoring of the MB molecule on both surfaces was carried out by means of two protons in a bidentate chelating (BC) adsorption model. The high adsorption energy of the MB dye on the $ZnTiO_3$ surface shows the potential value of using this mixed oxide as a dye adsorbent for several technological and environmental applications.

Keywords: DFT; $ZnTiO_3$; TiO_2; methylene blue; adsorption

Citation: Jaramillo-Fierro, X.; Capa, L.F.; Medina, F.; González, S. DFT Study of Methylene Blue Adsorption on $ZnTiO_3$ and TiO_2 Surfaces (101). *Molecules* **2021**, *26*, 3780. https://doi.org/10.3390/molecules26133780

Academic Editor: Monika Wawrzkiewicz

Received: 25 May 2021
Accepted: 19 June 2021
Published: 22 June 2021

Publisher's Note: MDPI stays neutral with regard to jurisdictional claims in published maps and institutional affiliations.

Copyright: © 2021 by the authors. Licensee MDPI, Basel, Switzerland. This article is an open access article distributed under the terms and conditions of the Creative Commons Attribution (CC BY) license (http://creativecommons.org/licenses/by/4.0/).

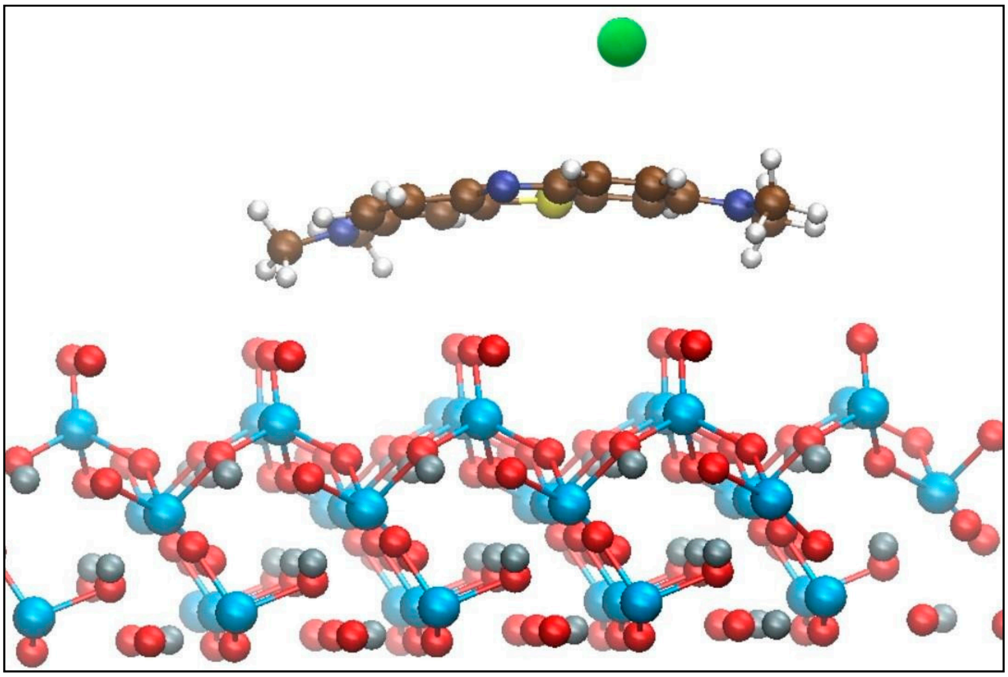

Figure S1. Aromatic ring of MB bent slightly on the $ZnTiO_3$ surface (101)

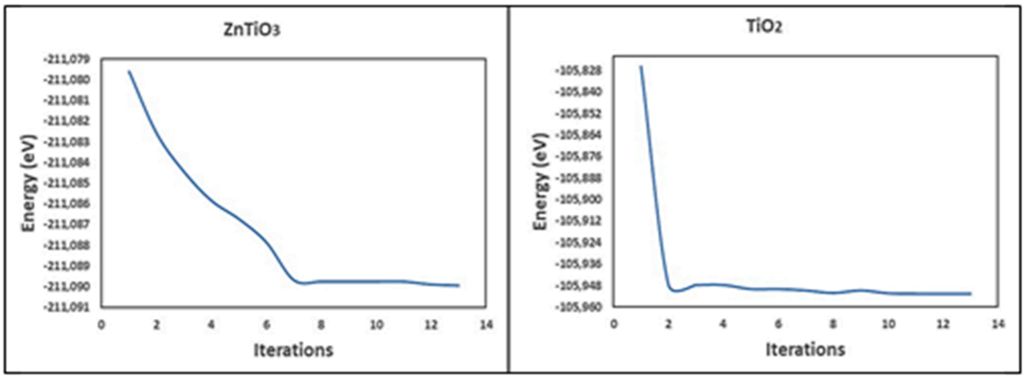

Figure S2. Optimization energies of (**a**) ZnTiO3 and (**b**) TiO2.

Table S1. Coordinates of the optimized $ZnTiO_3$ and TiO_2 structures.

ZnTiO3	Element	Coordinates X	Y	Z
a = 5.14797	O 1	0.0436517776555294	0.3434519311655431	0.0618327064818351
b = 5.14797	O 2	0.6565480388344616	0.7001998274899882	0.0618327064818351
c = 13.93750	O 3	0.2998001425100092	0.9563482113444732	0.0618327064818351
α = 90.0000	O 4	0.6565480388344616	0.9563482113444732	0.5618327214818327
β = 90.0000	O 5	0.2998001425100092	0.3434519311655431	0.5618327214818327
γ = 120.0000	O 6	0.0436517776555294	0.7001998274899882	0.5618327214818327
	O 7	0.7103184156555318	0.6767852741655427	0.3951660354818335
	O 8	0.3232147258344573	0.0335331784899886	0.3951660354818335
	O 9	0.9664667985100132	0.2896815543444728	0.3951660354818335
	O 10	0.3232147258344573	0.2896815543444728	0.8951660354818335
	O 11	0.9664667985100132	0.6767852741655427	0.8951660354818335
	O 12	0.7103184156555318	0.0335331784899886	0.8951660354818335
	O 13	0.3769851026555275	0.0101185981655372	0.7284993484818306
	O 14	0.9898814118344565	0.3668665144899912	0.7284993484818306

	O 15	0.6331334855100089	0.6230148973444725	0.7284993484818306
	O 16	0.9898814118344565	0.6230148973444725	0.2284993784818331
	O 17	0.6331334855100089	0.0101185981655372	0.2284993784818331
	O 18	0.3769851026555275	0.3668665144899912	0.2284993784818331
	Zn 1	0.0000000000000000	-0.0000000000000000	0.2790581887331681
	Zn 2	0.0000000000000000	-0.0000000000000000	0.7790581887331682
	Zn 3	0.6666666870000029	0.3333333429999996	0.6123915017331654
	Zn 4	0.6666666870000029	0.3333333429999996	0.1123915247331636
	Zn 5	0.3333333429999996	0.6666666870000029	0.9457248747331675
	Zn 6	0.3333333429999996	0.6666666870000029	0.4457248457331685
	Ti 1	0.0000000000000000	-0.0000000000000000	0.9988437118213425
	Ti 2	0.0000000000000000	-0.0000000000000000	0.4988437118213426
	Ti 3	0.6666666870000029	0.3333333429999996	0.3321770548213422
	Ti 4	0.6666666870000029	0.3333333429999996	0.8321770248213396
	Ti 5	0.3333333429999996	0.6666666870000029	0.6655103988213453
	Ti 6	0.3333333429999996	0.6666666870000029	0.1655103838213406
**TiO2**	**Element**		**Coordinates**	
		**X**	**Y**	**Z**
a = 3.82060	Ti 1	-0.0000000000000000	0.0000000000000000	0.0000000000000000
b = 3.82060	Ti 2	0.5000000000000000	0.5000000000000000	0.5000000000000000
c = 9.69652	Ti 3	0.0000000000000000	0.5000000000000000	0.2500000000000000
α = 90.0000	Ti 4	0.5000000000000000	-0.0000000000000000	0.7500000000000000
β = 90.0000	O 1	-0.0000000000000000	0.0000000000000000	0.2066838593974745
γ = 90.0000	O 2	0.5000000000000000	0.5000000000000000	0.7066838893974697
	O 3	-0.0000000000000000	0.5000000000000000	0.4566838593974745
	O 4	0.5000000000000000	0.0000000000000000	0.9566838893974697
	O 5	0.5000000000000000	-0.0000000000000000	0.5433161106025303
	O 6	0.0000000000000000	0.5000000000000000	0.0433161406025255
	O 7	0.5000000000000000	0.5000000000000000	0.2933161406025255
	O 8	0.0000000000000000	-0.0000000000000000	0.7933161106025303

Table S2. Bader`s charge analysis of the Methylene Blue molecule.

	**MB**				**MB Absorbed on $ZnTiO_3$**				**MB Absorbed on TiO_2**			
**Atom**	**X**	**Y**	**Z**	**Charge(-*e*)**	**X**	**Y**	**Z**	**Charge(-*e*)**	**X**	**Y**	**Z**	**Charge(-*e*)**
CL	6.6723	5.4913	18.1256	3.9473	5.7841	6.8665	18.8427	4.0377	8.5502	6.3036	37.8262	4.0165
C	6.6883	6.9248	18.2492	2.9801	5.4537	8.2487	19.0343	2.9038	7.1274	6.1282	37.9076	2.9116
C	6.8113	7.7022	17.0778	3.7228	5.5389	9.1145	17.9135	3.8886	6.3672	5.9423	36.7213	3.9439
C	6.8381	4.9031	16.9023	4.0263	6.1899	6.4038	17.6162	4.0043	9.0968	6.2958	36.5683	3.9895
C	6.9903	7.1050	15.8404	4.4306	5.9565	8.6387	16.6753	4.2272	6.9541	5.9232	35.4362	4.1712
C	7.0214	5.6667	15.7039	2.6235	6.3054	7.2586	16.4816	2.6376	8.4054	6.0825	35.3271	2.6542
C	7.4012	7.0194	13.1881	4.2267	6.6400	8.8226	14.0368	4.2430	7.1262	5.6325	32.7208	4.1458
C	7.6017	7.5508	11.9115	3.9548	6.8412	9.4525	12.8237	3.9190	6.6611	5.3793	31.4157	4.0569
C	7.7943	6.7163	10.7922	2.9653	7.3367	8.7318	11.6975	2.7809	7.5400	5.2562	30.3197	2.9217
C	7.7791	5.2961	10.9951	3.9989	7.6129	7.3252	11.8614	4.102	8.9224	5.3489	30.5791	3.9441
C	7.5841	4.7715	12.2459	3.9766	7.3997	6.7069	13.0583	3.8457	9.4304	5.5405	31.8646	4.0569
C	7.3901	5.5967	13.3925	2.632	6.9072	7.4076	14.2087	2.5409	8.5204	5.7274	32.9874	2.6496
C	8.0476	8.6800	9.3573	3.3209	7.2135	10.7579	10.3305	3.3619	5.9685	4.1045	28.7949	3.2969
C	8.1623	6.3644	8.3904	3.2358	8.1032	8.6179	9.3682	3.4622	7.6270	5.7136	27.888	3.4016
C	6.3617	6.7509	20.6746	3.2311	5.0669	7.8474	21.4312	3.3437	7.3591	6.2087	40.3865	3.3551
C	6.6204	8.9880	19.5700	3.3401	4.6740	10.1103	20.4200	3.2829	5.0623	5.8367	39.2665	3.7745
C	7.1937	8.1669	14.4764	5.6016	6.0469	9.7703	15.3638	5.7591	5.9774	5.7813	34.0176	5.7675

S	7.2001	4.9769	14.5721	7.7963	6.7432	6.7090	15.3311	7.8003	9.1383	5.9281	34.1814	7.7280
N	7.9923	7.2378	9.5422	7.4915	7.5403	9.3424	10.5148	7.4025	7.1210	4.9650	29.0625	7.4757
N	6.6024	7.5282	19.4673	7.4619	5.0720	8.7171	20.2557	7.4840	6.5601	6.1084	39.1715	7.0200
N	7.8071	10.6069	16.2128	7.7365	7.4174	9.9721	25.4704	7.6694	11.3226	6.5800	40.5672	7.5771
H	6.5464	4.8632	19.0063	0.9519	5.7058	6.1701	19.6745	0.9410	9.2393	6.4583	38.7013	0.8427
H	6.8463	3.8182	16.793	0.9123	6.4397	5.3533	17.4678	0.9051	10.2034	6.3392	36.4626	0.9552
H	6.8474	8.8004	17.1014	0.8673	5.2899	10.1698	18.0158	0.9714	5.2548	5.9168	36.7093	0.9938
H	7.6063	8.6347	11.8008	0.9618	6.6248	10.5151	12.7307	0.9768	5.5971	5.3471	31.2078	0.9224
H	7.9277	4.6224	10.1538	0.9834	7.9801	6.7477	11.0159	0.9303	9.6273	5.1718	29.7369	0.9330
H	7.5780	3.6933	12.4092	0.9595	7.5939	5.6415	13.1844	0.9355	10.4563	5.5461	32.1510	0.8849
H	8.2348	8.9005	8.3018	0.9762	7.3761	11.0287	9.28130	0.8688	6.2252	3.2788	28.0652	0.9363
H	7.0996	9.1625	9.6475	0.9452	6.1601	10.9473	10.5846	0.9927	5.6256	3.6424	29.726	0.9630
H	8.8571	9.1316	9.9535	0.9439	7.8548	11.3926	10.9641	0.9680	5.0539	4.6016	28.4023	0.9802
H	9.0590	5.7301	8.4852	0.9984	9.0775	8.1775	9.6186	0.9366	8.3192	6.4883	28.2325	0.9457
H	7.2886	5.7092	8.2442	0.9748	7.4344	7.8141	9.0297	0.9029	8.1160	4.9767	27.2684	0.9655
H	8.2768	6.9802	7.4928	0.9921	8.2389	9.3251	8.5457	0.9121	6.7832	6.1443	27.3016	0.9375
H	7.1440	5.9919	20.827	0.9953	6.0342	7.3406	21.5525	0.9403	7.8858	7.2124	40.3901	0.9220
H	6.3730	7.4256	21.5362	0.9362	4.8969	8.4559	22.3239	0.9438	6.7805	6.1236	41.3176	0.9454
